# Association Between Human Papillomavirus Infection Among Pregnant Women and Preterm Birth

**DOI:** 10.1001/jamanetworkopen.2021.25308

**Published:** 2021-09-15

**Authors:** Joseph Niyibizi, Marie-Hélène Mayrand, François Audibert, Patricia Monnier, Paul Brassard, Louise Laporte, Julie Lacaille, Monica Zahreddine, Marie-Josée Bédard, Isabelle Girard, Diane Francoeur, Ana Maria Carceller, Jacques Lacroix, William Fraser, François Coutlée, Helen Trottier

**Affiliations:** 1 Department of Social and Preventive Medicine, Université de Montréal, Montreal, Québec, Canada; 2 Centre de Recherche du Centre Hospitalier Universitaire Sainte-Justine, Université de Montréal, Montreal, Québec, Canada; 3 Department of Obstetrics and Gynecology, Université de Montréal, Montreal, Québec, Canada; 4 Centre de Recherche du Centre Hospitalier de l’Université de Montréal, Montreal, Québec, Canada; 5 Department of Obstetrics and Gynecology, Centre Hospitalier Universitaire Sainte-Justine, Université de Montréal, Montreal, Québec, Canada; 6 Department of Obstetrics and Gynecology, McGill University, Montreal, Québec, Canada; 7 Research Institute of the McGill University Health Center, Montreal, Québec, Canada; 8 Division of Clinical Epidemiology, McGill University, Montreal, Québec, Canada; 9 Department of Obstetrics and Gynecology, St-Mary’s Hospital Center, Montreal, Québec, Canada; 10 Department of Pediatrics, Centre Hospitalier Universitaire Sainte-Justine, Université de Montréal, Montreal, Québec, Canada; 11 Department of Obstetrics and Gynecology, Centre de Recherche du Centre Hospitalier Universitaire de Sherbrooke, Sherbrooke, Québec, Canada; 12 Département Clinique de Médecine de Laboratoire, Service de Biologie Moléculaire, Centre Hospitalier de l’Université de Montréal, Montréal, Québec, Canada; 13 Département de Médecine, Service d’Infectiologie, Centre Hospitalier de l’Université de Montréal, Montréal, Québec, Canada; 14 Département de Microbiologie, Infectiologie et Immunologie, Université de Montréal, Montreal, Québec, Canada

## Abstract

**Importance:**

Preterm birth remains a leading cause of perinatal mortality and lifelong morbidity worldwide. The cause of most preterm births is unknown, although several infectious processes have been implicated.

**Objective:**

To assess whether human papillomavirus (HPV) infection, a frequent infection among women of childbearing age, is associated with preterm birth.

**Design, Setting, and Participants:**

The prospective HERITAGE cohort study was conducted at 3 academic hospitals in Montreal, Québec, Canada, among 899 pregnant women recruited between November 8, 2010, and October 16, 2016. Follow-up was completed on June 15, 2017. Statistical analysis was conducted from February 6, 2020, to January 21, 2021.

**Exposures:**

Vaginal HPV DNA detection in the first and third trimesters of pregnancy and placental HPV infection.

**Main Outcomes and Measures:**

The main outcome was preterm birth (defined as a live birth or stillbirth between 20 weeks and 0 days and 36 weeks and 6 days of gestation). The association between HPV DNA detection and preterm birth was measured using logistic regression. Odds ratios (ORs) and 95% CIs were adjusted by inverse probability of treatment weights of the propensity score.

**Results:**

The study included 899 women (mean [SD] age, 31.3 [4.6] years [range, 19-47 years]) with singleton pregnancies. A total of 378 women (42.0%) had HPV DNA detected in vaginal samples collected during the first trimester, and it was detected in 91 of 819 placentas (11.1%) at delivery. Fifty-five participants experienced preterm birth (38 spontaneous and 17 medically indicated). Persistent vaginal HPV-16/18 detection was significantly associated with all preterm births (adjusted OR [aOR], 3.72; 95% CI, 1.47-9.39) and spontaneous preterm births (aOR, 3.32; 95% CI, 1.13-9.80), as was placental HPV infection (all preterm births: aOR, 2.53; 95% CI, 1.06-6.03; spontaneous preterm births: aOR, 2.92; 95% CI, 1.09-7.81). Results were similar when restricting the analysis to participants without a history of cervical intraepithelial neoplasia treatment.

**Conclusions and Relevance:**

The study’s results suggest that persistent HPV-16/18 infection is associated with an increased risk of preterm birth, independent of cervical treatment. Future studies should investigate the association of HPV vaccination and vaccination programs with the risk of preterm birth.

## Key Points

**Question:**

Is human papillomavirus (HPV) infection during pregnancy associated with preterm delivery?

**Findings:**

In this multicenter cohort study of 899 pregnant women, persistent HPV-16/18 infection during pregnancy was significantly associated with preterm birth.

**Meaning:**

If the identified association between HPV infection during pregnancy and preterm delivery is causal, HPV vaccination may reduce preterm birth and the associated burden.

## Teaser

This cohort study assesses whether human papillomavirus infection, a frequent infection among women of childbearing age, is associated with preterm birth.

## Introduction

Preterm birth, the etiology of which is often unknown, remains one of the leading causes of perinatal mortality and lifelong morbidity worldwide.^1^ Genital tract viral infections may alter the protection offered by the cervical epithelium and facilitate ascending bacterial infection, causing preterm labor.^2^ Viral infections may also directly inhibit trophoblast function and disrupt the fetal-placental-maternal immune system, increasing the risk of preterm labor and birth.^3,4^

Human papillomavirus (HPV) is the most common viral infection of the genital tract.^5^ In Canada, the age group with the highest prevalence of HPV infection (15-30 years) overlaps with the age group with the highest birth rates (25-34 years).^6^ In vitro studies^7^ and animal models^8^ have implicated HPV infections in several adverse pregnancy outcomes. However, clinical studies have yielded mixed results regarding the association between HPV and preterm birth, possibly owing to HPV exposure misclassification, detection of HPV at inappropriate time points, and insufficient control for confounding.^9^ Given the high prevalence of HPV infection among women of childbearing age, the mechanistic plausibility of an association between preterm birth and HPV infection, the important burden of preterm birth, and the availability of effective HPV vaccines as a preventive method, confirmation of this association is of utmost interest. Therefore, we prospectively assessed whether vaginal HPV infections during pregnancy and placental HPV at birth were independently associated with preterm birth.

## Methods

### Design, Study Population, and Collection of Samples

We conducted a prospective cohort study (the HERITAGE study) enrolling 1052 pregnant women from 3 university-affiliated health care centers in Montreal, Québec, Canada (Centre Hospitalier Universitaire Sainte-Justine, Centre Hospitalier de l’Université de Montréal, and Saint-Mary’s Hospital Center) between November 8, 2010, and October 16, 2016. The date of the last delivery was June 15, 2017. The study design and procedures have been described previously.^10^ In brief, pregnant women of at least 18 years of age were recruited during the first trimester of pregnancy. Women who were HIV positive or unable to provide written consent were not eligible for the HERITAGE cohort study. For this analysis, we excluded women with multiple pregnancies (twins or more), women who had spontaneous or induced abortions, and women with a history of cervico-isthmic incompetency who underwent a prophylactic cerclage in the first trimester. The study folllowed the Strengthening the Reporting of Observational Studies in Epidemiology (STROBE) reporting guideline. The institutional ethical and research review boards of the Centre Hospitalier Universitaire Sainte-Justine, the Centre Hospitalier de l’Université de Montréal, and the Saint-Mary’s Hospital Center approved this study. Each participant provided written informed consent.

At recruitment, participants provided a self-collected vaginal sample for genotype-specific HPV testing. Participants who tested positive for HPV DNA at the first trimester visit provided additional swab samples for genotype-specific HPV DNA testing at 32 to 35 weeks’ gestation. Placental swab samples and biopsy specimens were collected immediately after birth from the fetal side of the placenta and the maternal side of the placenta after a standardized procedure designed to minimize the risk of contamination.^10^

Sociodemographic status, medical and sexual history, and tobacco and alcohol consumption were documented at baseline. Self-identified ethnic origin was collected and provided for the sole purpose of describing the study population. Pregnancy history and delivery information were extracted from electronic medical records.

### HPV DNA Testing

Human papillomavirus detection and genotyping were performed with the Linear Array genotyping assay (Roche Molecular Systems) detecting 36 mucosal HPV genotypes, including genotypes 6, 11, 16, 18, 26, 31, 33, 34, 35, 39, 40, 42, 44, 45, 51, 52, 53, 54, 56, 58, 59, 61, 62, 66, 67, 68, 69, 70, 71, 72, 73, 81, 82, 83, 84, and 89. In this study, HPV genotypes 16, 18, 31, 33, 35, 39, 45, 51, 52, 56, 58, 59, 66, 68, 73, and 82 were considered as high-risk HPV genotypes, and HPV genotypes 6, 11, 26, 34, 40, 42, 44, 53, 54, 61, 62, 67, 69, 70, 71, 72, 81, 83, 84, and 89 were considered as low-risk HPV genotypes.^11,12,13^

### Definition of Preterm Birth

*Preterm birth* was defined as a live birth or stillbirth between 20 weeks and 0 days and 36 weeks and 6 days of gestation.^14^ First-trimester ultrasonography is part of routine prenatal care in the recruiting centers and was used to confirm gestational age. The outcome of our primary analysis was overall preterm birth, which included all births occurring between 20 weeks and 0 days and 36 weeks and 6 days of gestation. We also analyzed HPV infection as a risk factor for spontaneous preterm births specifically. This category included only births after spontaneous onset of labor or preterm premature rupture of membranes. Participants presenting with cervical dilation in the second trimester requiring emergency cerclage (n = 5) were included in the spontaneous preterm birth category irrespective of gestational age at delivery.

### Definition of Exposure

Exposure to HPV was conceptualized as (1) vaginal HPV infection detected in the first trimester; (2) persistent HPV infection, defined as vaginal HPV infection detected in both the first and third trimesters; and (3) placental HPV infection. We first categorized vaginal HPV infection detected in the first trimester as a binary variable: negative or positive for any HPV genotype. Subsequently, we constructed a 4-level variable with mutually exclusive categories: negative for HPV, positive for low-risk HPV only, positive for high-risk HPV but not genotypes 16/18, and positive for HPV-16 or HPV-18. *Persistent HPV infection* was defined as a variable with 5 mutually exclusive categories: negative for HPV, transient HPV infection with any type (positive at the first trimester only), persistent low-risk HPV only, persistent high-risk HPV but not genotypes 16/18, and persistent HPV-16/18 at both the first and third trimesters. For placental HPV infection, we used a binary categorization: negative or positive for any HPV genotype after we pooled all HPV DNA testing results obtained from swab samples and biopsy specimens.

### Missing Data

Rates of missing values were lower than 1% for all variables except pregnancy-induced hypertensive disorders (10 of 899 [1.1%]), alcohol use (13 of 899 [1.4%]), gestational diabetes (21 of 899 [2.3%]), and history of treatment for cervical intraepithelial neoplasia (73 of 899 [8.1%]). Missingness was not associated with the observed values of the outcome or the exposure. Thus, we assumed that a complete case analysis would not have led to bias.^15^ However, 113 participants (12.6%) had at least 1 variable with a missing value. Therefore, for the sake of precision, we imputed missing data by the mean if the missing value was a continuous variable or by the mode if the missing value was a categorical variable.

### Statistical Analysis

#### Primary Analysis

Statistical analysis was conducted from February 6, 2020, to January 21, 2021. To assess the association between HPV infection and preterm birth, odds ratios (ORs) and their 95% CIs were estimated using unconditional logistic regression. To control for confounding, adjusted ORs (aORs) and their 95% CIs were obtained using inverse propensity treatment weights (IPTW).^16^ We estimated a propensity score of any HPV infection during the first trimester and a propensity score of any placental HPV infection. The selection of factors associated with propensity scores was based on a priori knowledge of risk factors for preterm birth, regardless of whether or not they were associated with HPV infection.^17^ The following factors that have been consistently associated with preterm births across several studies were used to estimate propensity scores: maternal age (years), ethnic origin (White vs others [as defined in Table 1]), completed education (in years), smoking at enrollment (yes or no), number of days when alcohol was consumed since the beginning of the pregnancy (none, 1-4 days, or ≥5 days), history of preterm birth (multiparous without preterm birth, multiparous with preterm birth, or nulliparous), history of cervical treatment for intraepithelial neoplasia (yes or no), urinary tract or genital infections (yes or no), gestational diabetes (yes or no), and pregnancy-induced hypertensive disorders (yes or no).^18,19,20^ We assessed the balance of covariates using standardized differences between women with and women without HPV before and after weighting data by IPTW. A standardized difference of less than 0.10 was considered as sufficient balance.^16^ A balance of covariates according to the vaginal HPV status at first trimester and placental HPV at birth was reached after weighting by IPTW (eFigure 1 and eFigure 2 in Supplement 1).

**Table 1.** Maternal Characteristics According to HPV Infection During the First Trimester

Characteristic	Women, No./total No. (%)		
	HPV negative (n = 521)	HPV positive (n = 378)	Total (N = 899)^a^
Maternal characteristics at recruitment			
Age, mean (SD) [range], y	31.7 (4.6) [19-43]	30.7 (4.7) [19-47]	31.3 (4.6) [19-47]
Gestational age at enrollment, median (IQR), completed wk	11 (10-12)	11 (10-12)	11 (10-12)
Completed years of education, median (IQR)	17 (15-18)	16 (14-18)	17 (14-18)
Ethnic origins			
White	360/520 (69.2)	289/378 (76.5)	649/898 (72.3)
Arabic-West Asian	52/520 (10.0)	16/378 (4.2)	68/898 (7.6)
Latin American	28/520 (5.4)	24/378 (6.3)	52/898 (5.8)
Native African	24/520 (4.6)	14/378 (3.7)	38/898 (4.2)
East Asian	11/520 (2.1)	4/378 (1.1)	15/898 (1.7)
African American	2/520 (0.4)	3/378 (0.8)	5/898 (0.6)
South Asian	4/520 (0.8)	0	4/898 (0.4)
Indigenous people	0	1/378 (0.3)	1/898 (0.1)
Others^b^	39/520 (7.5)	27/378 (7.1)	66/898 (7.3)
Current smoker	32/518 (6.2)	57/377 (15.1)	89/895 (9.9)
Alcohol consumption since pregnant, d^c^			
None	358/512 (69.9)	209/374 (55.9)	567/886 (64.0)
1-4	112/512 (21.9)	119/374 (31.8)	231/886 (26.1)
≥5	42/512 (8.2)	46/374 (12.3)	88/886 (9.9)
At least 1 new sexual partner in the last year	16/518 (3.1)	50/377 (13.3)	66/895 (7.4)
Nulliparous	209/519 (40.3)	199/377 (52.8)	408/896 (45.5)
History of preterm birth among parous women	66/310 (21.3)	27/178 (15.2)	93/488 (19.1)
Received at least 1 dose of an HPV vaccine	40/490 (8.2)	39/357 (10.9)	79/847 (9.3)
History of treatment for cervical intraepithelial neoplasia^d^	22/480 (4.6)	37/346 (10.7)	59/826 (7.1)
Pregnancy disorders			
Gestational diabetes	73/510 (14.3)	44/368 (12.0)	117/878 (13.3)
Pregnancy-induced hypertensive disorders	24/519 (4.6)	14/370 (3.8)	38/889 (4.3)
Urinary tract or genital infection^e^	13/520 (2.5)	10/377 (2.7)	23/897 (2.6)

Abbreviations: HPV, human papillomavirus; IQR, interquartile range.

^a^ Total percentage may not add up to 100% owing to missing values.

^b^ Participants who self-identified as being in 2 different ethnic groups were assigned to the others group.

^c^ Number of days participants reported consuming at least 1 alcoholic beverage since the beginning of pregnancy.

^d^ Methods of treatment for cervical intraepithelial neoplasia consisted of excisional treatments (11 HPV-negative women and 26 HPV-positive women) and ablative treatments (7 HPV-negative women and 7 HPV-positive women). The type of treatment was unknown for 8 participants (4 HPV-negative women and 4 HPV-positive women).

^e^ Urinary tract or genital infection includes cystitis (8 HPV-negative women and 6 HPV-positive women), bacterial vaginosis (1 HPV-negative woman and 1-HPV positive woman), active herpetic lesion (4 HPV-negative women and 1 HPV-positive woman), and nonspecified urinary tract or genital infection (2 HPV-positive women).

All *P* values were from 2-sided tests, and results were deemed statistically significant at *P* < .05. All analyses were performed using Stata/SE, version 14.2 (StataCorp LLC).

#### Subgroup Analyses

To assess the robustness of our findings, we conducted 3 a priori–specified exploratory subgroup analyses. First, because a history of preterm birth can be considered a marker of unknown recurrent or chronic risk factors,^18^ we stratified analysis according to history of preterm birth. Moreover, considering that cervical treatment for intraepithelial neoplasia in and of itself increases the risk of preterm birth by altering the physical integrity of the cervix,^19^ we compared women with a history of cervical intraepithelial neoplasia treatment and women without. Finally, because smoking is hypothosized to augment the effect of HPV in precancerous and cancerous pathologic conditions,^21^ we explored whether the association between HPV infection and preterm birth differed between smokers and nonsmokers.

## Results

### Participants

**Figure 1.**
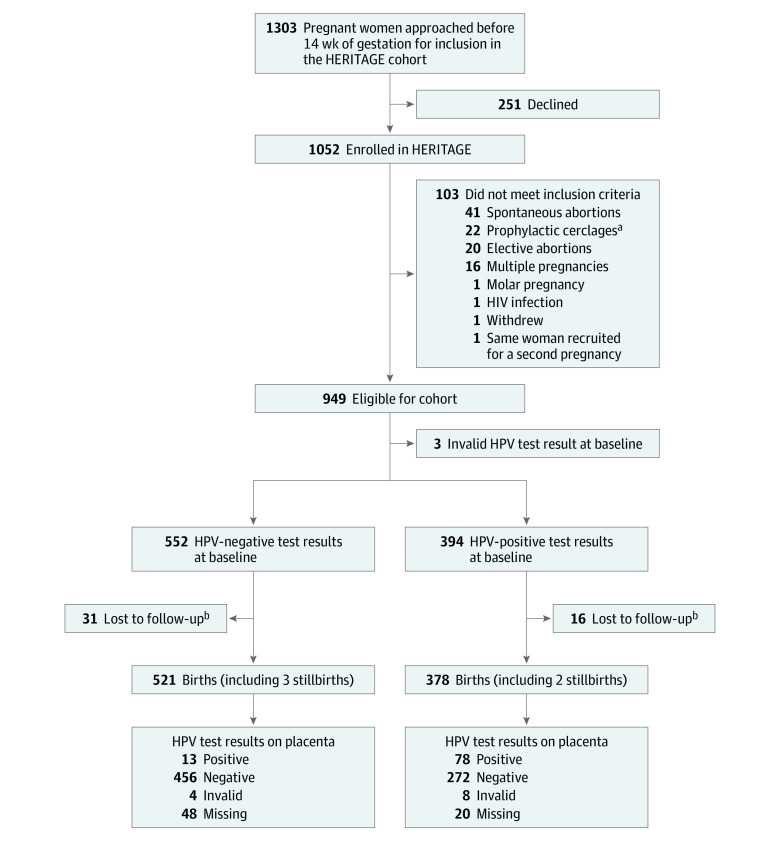
Study Flow Diagram HPV indicates human papillomavirus. ^a^A total of 22 participants underwent first-trimester prophylactic cerclage because of a history of cervico-isthmic insufficiency in a previous pregnancy. ^b^Withdrawn or deliveries at nonparticipating site.

After excluding HERITAGE cohort participants who lost or terminated their pregnancy, those with a multiple pregnancy, and women with a history of cervico-isthmic insufficiency who underwent a first-trimester prophylactic cerclage, the final sample included 899 women with singleton pregnancies and a valid first-trimester HPV DNA test result (Figure 1).

Table 1 summarizes the characteristics of the participants. Overall, the mean (SD) age at enrollment was 31.3 (4.6) years, and most of the study participants were White (649 [72.3%]), had a university education (median completed years of education, 17 [interquartile range, 14-18 years]), and did not smoke (810 [90.1%]) or drink alcohol (567 [64.0%]) at enrollment. A total of 408 women (45.5%) were nulliparous, but only 79 (9.3%) had received the HPV vaccine, and a similarly low proportion of women (59 [7.1%]) had a history of treatment for cervical intraepithelial neoplasia. Only 23 women (2.6%) had a genital or urinary tract infection during pregnancy. Participants were tested for genital *Chlamydia trachomatis* and *Neisseria gonorrhoea* in the first trimester as part of routine prenatal care, and none had positive test results. Compared with participants who had negative HPV test results (n = 521), those who had positive HPV test results (n = 378) were more likely to self-identify as White (289 [76.5%] vs 360 [69.2%]), smoke (57 [15.1%] vs 32 [6.2%]), have had a new sexual partner in the last year (50 [13.3%] vs 16 [3.1%]), have had a history of treatment for cervical intraepithelial neoplasia (37 [10.7%] vs 22 [4.6%]), and be nulliparous (199 [52.8%] vs 209 [40.3%]).

### HPV Infection

Overall, 378 participants (42.0%) had positive HPV test results at the first-trimester recruitment visit, among whom 252 participants (28.0%) were infected with at least 1 high-risk HPV genotype and 167 (18.6%) were infected with more than 1 HPV genotype (Table 2). Most participants with positive HPV test results during the first trimester also had positive HPV test results in the third trimester (68.3% [258 of 378]). Of the 258 women with positive HPV test results during the third trimester, 63 (24.4%) were infected with a new HPV genotype. Only 91 of 819 placentas (11.1%) that were sampled harbored HPV DNA.

**Table 2.** Characteristics of HPV Infection

Characteristic	Total sample, No. (%) (N = 899)
**Vaginal HPV infections**	
First trimester	
Any HPV infection	378 (42.0)
Single-genotype infection	211 (23.5)
Multiple-genotype infection	167 (18.6)
Low-risk HPV only	126 (14.0)
High-risk HPV other than HPV-16/18	186 (20.7)
HPV-16/18	66 (7.3)
First and third trimesters	
Any HPV, first trimester only	120 (13.3)
Low-risk HPV	92 (10.2)
High-risk HPV other than HPV-16/18	122 (13.6)
HPV-16/18	44 (4.9)
**Placental HPV infections (n = 819)** ^**a**^	
Any HPV infection	91 (11.1)
Single-genotype infection	65 (7.9)
Multiple-genotype infection	26 (3.2)
Low-risk HPV only	25 (3.1)
High-risk HPV other than HPV-16/18	41 (5.0)
HPV-16/18	25 (3.1)

Abbreviation: HPV, human papillomavirus.

^a^ Of the total sample, there were 12 invalid HPV DNA test results from placental samples and 68 missing placentas.

### HPV Infection and Preterm Birth

Overall, 55 participants experienced preterm birth, of which 38 were spontaneous and 17 were medically indicated. The median gestational age of preterm infants was 36.0 weeks (interquartile range, 34-36 weeks). Of those infants, 41 were born after 34 weeks of gestational age, 6 were born between 32 and 34 weeks, and 8 were born before 32 weeks. As shown in Table 3, the detection of vaginal HPV DNA (any genotype) during the first trimester was not associated with an increased risk of preterm birth (aOR, 1.39; 95% CI, 0.79-2.46). However, the detection of HPV-16/18 infection during the first trimester was associated with an increased risk of preterm birth (aOR, 2.55; 95% CI, 1.07-6.04). The persistence of HPV-16/18 infection between the first and third trimesters was associated with an increased risk of both overall preterm birth (aOR, 3.72; 95% CI, 1.47-9.39) and spontaneous preterm birth (aOR, 3.32; 95% CI, 1.13-9.80). Placental detection of any HPV DNA was also associated with all preterm births (aOR, 2.53; 95% CI, 1.06-6.03) and spontaneous preterm births (aOR, 2.92; 95% CI, 1.09-7.81). The Kaplan-Meier curves presented in eFigure 3 in Supplement 1 show the cumulative incidence of preterm birth according to HPV-16/18 persistence.

**Table 3.** Association Between HPV Infection and Preterm Birth

Exposure categories^a^	Outcome, No./total No. (%)	Odds ratio (95% CI)	
		Crude	Adjusted^b^
**All preterm births**			
Vaginal HPV, first trimester			
Negative	29/521 (5.6)	1 [Reference]	NA
Positive, any HPV	26/378 (6.9)	1.25 (0.72-2.16)	1.39 (0.79-2.46)
Vaginal HPV genotype groups, first trimester			
Negative	29/521 (5.6)	1 [Reference]	NA
Low-risk HPV only	7/126 (5.6)	1.00 (0.43-2.33)	1.30 (0.53-3.15)
High-risk HPV other than HPV-16/18	11/186 (5.9)	1.07 (0.52-2.18)	1.07 (0.50-2.26)
HPV-16/18	8/66 (12.1)	2.34 (1.02-5.36)	2.55 (1.07-6.04)
Vaginal HPV genotype groups, first and third trimesters			
Negative	29/521 (5.6)	1 [Reference]	NA
Any HPV, first trimester only	7/120 (5.8)	1.05 (0.45-2.46)	1.15 (0.47-2.82)
Low-risk HPV only	6/92 (6.5)	1.18 (0.48-2.93)	1.49 (0.57-3.91)
High-risk HPV other than HPV-16/18	6/122 (4.9)	0.88 (0.36-2.16)	0.84 (0.33-2.10)
HPV-16/18	7/44 (15.9)	3.21 (1.32-7.82)	3.72 (1.47-9.39)
Placental HPV^c^			
Negative	35/728 (4.8)	1 [Reference]	NA
Positive, any HPV	9/91 (9.9)	2.17 (1.01-4.68)	2.53 (1.06-6.03)
**Spontaneous preterm births**			
Vaginal HPV, first trimester			
Negative	22/514 (4.3)	1 [Reference]	NA
Positive, any HPV	16/368 (4.3)	1.02 (0.53-1.96)	1.06 (0.54-2.09)
Vaginal HPV genotype groups, first trimester			
Negative	22/514 (4.3)	1 [Reference]	NA
Low-risk HPV only	4/123 (3.2)	0.75 (0.25-2.22)	0.87 (0.29-2.64)
High-risk HPV other than HPV-16/18	7/182 (3.8)	0.89 (0.37-2.13)	0.85 (0.35-2.07)
HPV-16/18	5/63 (7.9)	1.93 (0.70-5.28)	2.06 (0.71-5.97)
Vaginal HPV genotype groups, first and third trimesters			
Negative	22/514 (4.3)	1 [Reference]	NA
Any HPV, first trimester only	4/117 (3.4)	0.79 (0.27-2.34)	0.81 (0.26-2.50)
Low-risk HPV only	3/89 (3.4)	0.78 (0.23-2.66)	0.80 (0.23-2.79)
High-risk HPV other than HPV-16/18	4/120 (3.3)	0.77 (0.26-2.28)	0.79 (0.26-2.38)
HPV-16/18	5/42 (11.9)	3.02 (1.08-8.44)	3.32 (1.13-9.80)
Placental HPV^c^			
Negative	24/717 (3.3)	1 [Reference]	NA
Positive, any HPV	7/89 (7.9)	2.46 (1.03-5.90)	2.92 (1.09-7.81)

Abbreviations: HPV, human papillomavirus; NA, not applicable.

^a^ All exposure categories are mutually exclusive.

^b^ Adjusted estimates obtained using propensity score–based inverse propensity treatment weights. The model of the propensity score of any HPV at the first trimester and any HPV in placenta included the following variables: maternal age (years), ethnic origin (White or other), completed education (years), smoking at enrollment (yes or no), total days of alcohol use since pregnant (none, 1-4 days, or ≥5 days), history of preterm birth (multiparous without preterm birth, multiparous with preterm birth, or nulliparous), history of cervical intraepithelial neoplasia treatment (yes or no), urinary tract or genital infections (yes or no), gestational diabetes (yes or no), and pregnancy-induced hypertensive disorders (yes or no).

^c^ Total of 80 missing (11 preterm births and 69 term births). For every placenta, 6 swab samples and 4 biopsy specimens (of the peripheric and central zone) of the maternal and fetal side were collected and underwent HPV testing. Results were pooled for statistical analysis.

### Subgroup Analyses

**Figure 2.**
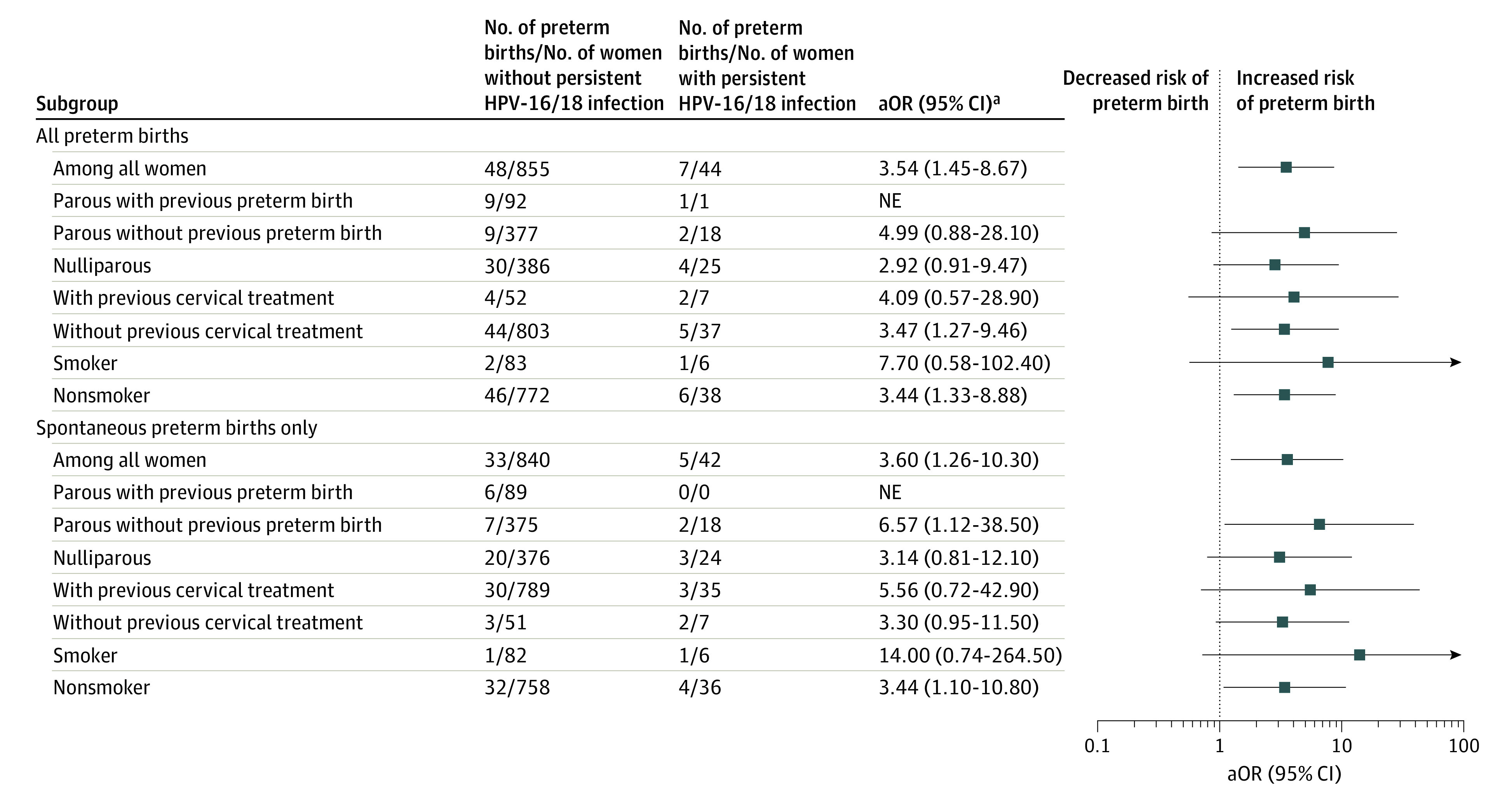
Subgroup Analyses for Human Papillomavirus 16/18 (HPV-16/18) Persistent Infection vs No HPV-16/18 Persistent Infection NE indicates nonestimable. ^a^Adjusted odds ratios (aORs) by inverse propensity treatment weights of the propensity score of any HPV at the first trimester, excluding the variable for which we stratified. Persistent HPV-16/18 infection was defined as a positive test result for HPV-16 or HPV-18 at the first trimester and third trimester. No persistent HPV-16/18 infection was defined as a negative test result for any HPV at both trimesters, any HPV transient infection at the first and third trimesters, or any persistent HPV infection other than HPV-16/18.

Given the association between persistent HPV-16/18 and preterm birth, we explored how this association may vary depending on an a priori risk of preterm birth. We compared women with and without persistent HPV-16/18 according to history of preterm birth, history of cervical intraepithelial neoplasia treatment, and tobacco smoking (Figure 2). Although our power to reach firm conclusions was limited by the small number of events in several subgroups, aORs for the association between persistent HPV-16/18 infection and both all and spontaneous preterm births remained consistently elevated for each subgroup studied. The largely overlapping 95% CIs limit our interpretation of the results, but the relative risk estimates do not suggest that the association between HPV and preterm birth is limited to certain subgroups.

## Discussion

We found that, even in a population considered to be at low risk based on sociodemographic and sexual history characteristics, HPV infection is frequent in pregnancy, and most HPV infections detected in the first trimester persist to the third trimester. The prevalence of HPV infection in our study was higher than previously described in several cohorts that also used broad-spectrum, sensitive polymerase chain reaction–based methods to detect HPV on cervicovaginal specimens.^22,23,24^ However, a similar high HPV DNA detection rate was observed on vaginal specimens in a cohort of women attending college in the same city.^25^

We did not find an association between vaginal HPV detection (any genotype) in the first trimester of pregnancy and preterm birth. However, the persistence of vaginal HPV-16/18 infection during pregnancy and the detection of any HPV on a placental swab sample were independently associated with the occurrence of both all and spontaneous preterm births. In a recent meta-analysis, HPV infections or HPV-related cytologic abnormalities were associated with preterm births but to a lesser degree than what was found in this study (pooled OR, 1.50; 95% CI, 1.19-1.88; 19 studies).^9^ However, several studies included in the meta-analysis used abnormal Papanicolaou test results before or after pregnancy as a surrogate for HPV infection during pregnancy,^26,27,28,29,30,31^ some studies targeted only a limited number of HPV genotypes,^7,32,33^ and most studies did not take into account important confounders, such as age, previous treatment for cervical intraepithelial neoplasia, other genital infections, or other obstetrical risk factors.^7,26,34,35,36^ All of those factors may have been associated with a biased, lower estimate of the association between HPV infection and preterm birth.

Treatment for cervical dysplasia caused by high-risk HPV increases the risk of preterm birth.^19^ It was not possible, in ecological or database studies, to differentiate between the role of HPV infection in and of itself and that of treatment for cervical dysplasia caused by high-risk HPV.^37^ In our population, the prevalence of cervical treatment was low, making it unlikely that it could explain the association between HPV and preterm birth. Moreover, excluding women who had a history of treatment did not change our conclusion. Another challenge of studying this topic is that HPV infections are more frequent among women who have other risk factors for preterm birth, such as young age, tobacco smoking, other genital infection, and low socioeconomic status,^18,20,38^ and thus may act merely as a marker for a pregnancy at higher risk of preterm birth. Through our detailed questionnaires and available clinical data, we were able to control for such factors using IPTW.

We can only speculate as to the pathophysiological mechanism underlying the association between HPV infection and preterm birth, independent of treatment for cervical dysplasia. Human papillomavirus infection of the keratinized epithelium (skin) and squamous or glandular epithelia (such as the cervical epithelium) does not induce a measurable inflammatory reaction. However, HPV infection may be associated with overall changes in the vaginal microbiota, which in turn could trigger an immuno-inflammatory process leading to preterm birth. Further research should investigate this possibility in suitable animal models.

### Strengths and Limitations

This study has several strengths. The main strength of this study is its prospective design that allowed for the detection of HPV throughout the exposure window period (pregnancy), during which HPV can exert a direct detrimental effect.^4^ We also detected several HPV genotypes, which enabled us to measure the association for distinct HPV genotypes. Furthermore, we used a standardized sampling protocol to avoid placental contamination. On both sides of each placenta, we collected 3 swab samples (including 1 under the amniotic membrane) and 4 biopsy specimens. Our prevalence of placental HPV (11.1%) was similar to that reported by a Danish study using in situ hybridization to localize HPV within trophoblastic cells.^39^ Using the IPTW technique, we generated a weighted sample in which the distribution of all measured confounders was similar between the groups of women studied. Last, having a detailed history of cervical dysplasia treatment enabled us to focus on HPV infection among women who had not been treated in the past.

This study also has some limitations. First, although the IPTW adjustment allowed for the full control of many confounders, some residual confounding is still possible. However, given the fact that the most important known risk factors for preterm birth were measured and taken into account either through restriction or adjustment by IPTW and that subgroups analysis showed consistent associations between HPV and preterm birth, it seems unlikely that residual confounding would explain our findings. Second, owing to a small number of events, we could not reach a conclusion on whether or not HPV infection conferred a higher risk of preterm birth in subgroups of women with an elevated baseline risk. Despite our use of a strict, standardized protocol for handling and testing the placental specimens, contamination of the placenta during birth can never be completely ruled out as an explanation for HPV-positive placental test results. However, the fact that HPV was detected on biopsy specimens and under the amniotic membrane indicates that true placental infection is more likely. Finally, we acknowledge that the study population of HERITAGE has several characteristics making it at low risk of preterm birth and that our conclusions may not translate to higher-risk populations.

## Conclusion

This is the first report, to our knowledge, to prospectively observe that persistent HPV-16/18 infection in pregnancy and placental HPV infection significantly increase the risk of preterm birth above infections by other HPV genotypes and separately from a history of cervical dysplasia treatment. If confirmed in larger and more diverse populations, these findings would support a role for HPV vaccination programs in the reduction of the burden of preterm births. This positive association would likely be observed during the next decade at the population level in settings with high-coverage HPV vaccination programs, when cohorts of girls vaccinated before sexual debut reach childbearing age.

## Notes

Supplement 1. **eFigure 1.** Covariates Balance Before and After Weighting Data by IPTW of Propensity Score of Any HPV at First Trimester **eFigure 2.** Covariates Balance Before and After Weighting Data by IPTW of Propensity Score of Any Placental HPV **eFigure 3.** Kaplan-Meier Curves Presenting the Cumulative Incidence of Preterm Birth According to HPV16/18 Persistence During Pregnancy

Supplement 2. **Nonauthor Collaborators.** The HERITAGE Study Group Members
