# Supplementary material for: Association Between Human Papillomavirus Infection Among Pregnant Women and Preterm Birth
Source: JAMA Netw Open. 2021 Sep 15;4(9):e2125308. doi: 10.1001/jamanetworkopen.2021.25308 (PMC8444026; doi:10.1001/jamanetworkopen.2021.25308)
Supplement: Supplement 1. — eFigure 1. Covariates Balance Before and After Weighting Data by IPTW of Propensity Score of Any HPV at First Trimester eFigure 2. Covariates Balance Before and After Weighting Data by IPTW of Propensity Score of Any Placental HPV eFigure 3. Kaplan-Meier Curves Presenting the Cumulative Incidence of Preterm Birth According to HPV16/18 Persistence During Pregnancy [file jamanetwopen-e2125308-s001.pdf]

Supplementary Online Content

Niyibizi J, Mayrand MH, Audibert F, et al. Association between human papillomavirus infection among pregnant women and preterm birth. *JAMA Netw Open.* 2021;4(9):e2125308. doi:10.1001/jamanetworkopen.2021.25308

eFigure 1. Covariates Balance Before and After Weighting Data by IPTW of Propensity Score of Any HPV at First Trimester
eFigure 2. Covariates Balance Before and After Weighting Data by IPTW of Propensity Score of Any Placental HPV
eFigure 3. Kaplan-Meier Curves Presenting the Cumulative Incidence of Preterm Birth According to HPV16/18 Persistence During Pregnancy

This supplementary material has been provided by the authors to give readers additional information about their work.

© 2021 Niyibizi J et al. *JAMA Network Open.*

eFigure 1. Covariates Balance Before and After Weighting Data by IPTW of Propensity Score of any HPV at First Trimester

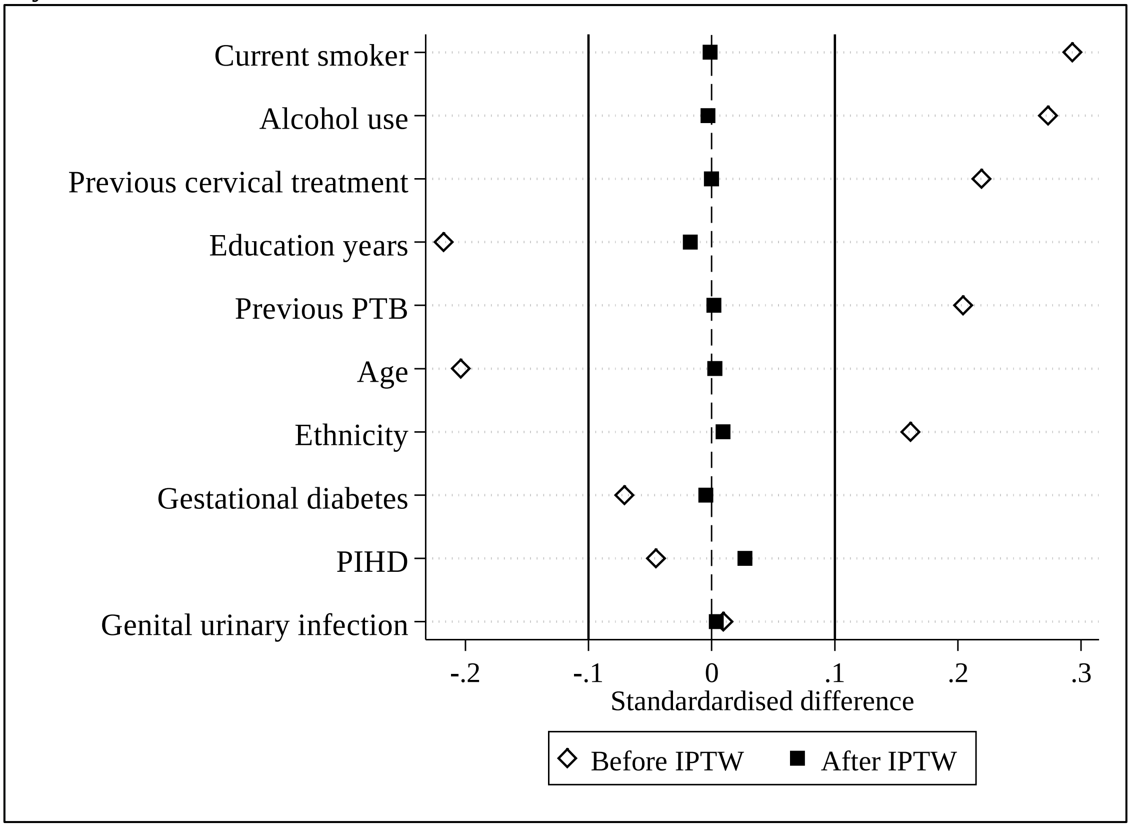

PTB: preterm birth; PIHD: pregnancy induced hypertensive disorders; IPTW: inverse probability treatment weighting

© 2021 Niyibizi J et al. *JAMA Network Open.*

eFigure 2. Covariates Balance Before and After Weighting Data by IPTW of Propensity Score of any Placental HPV

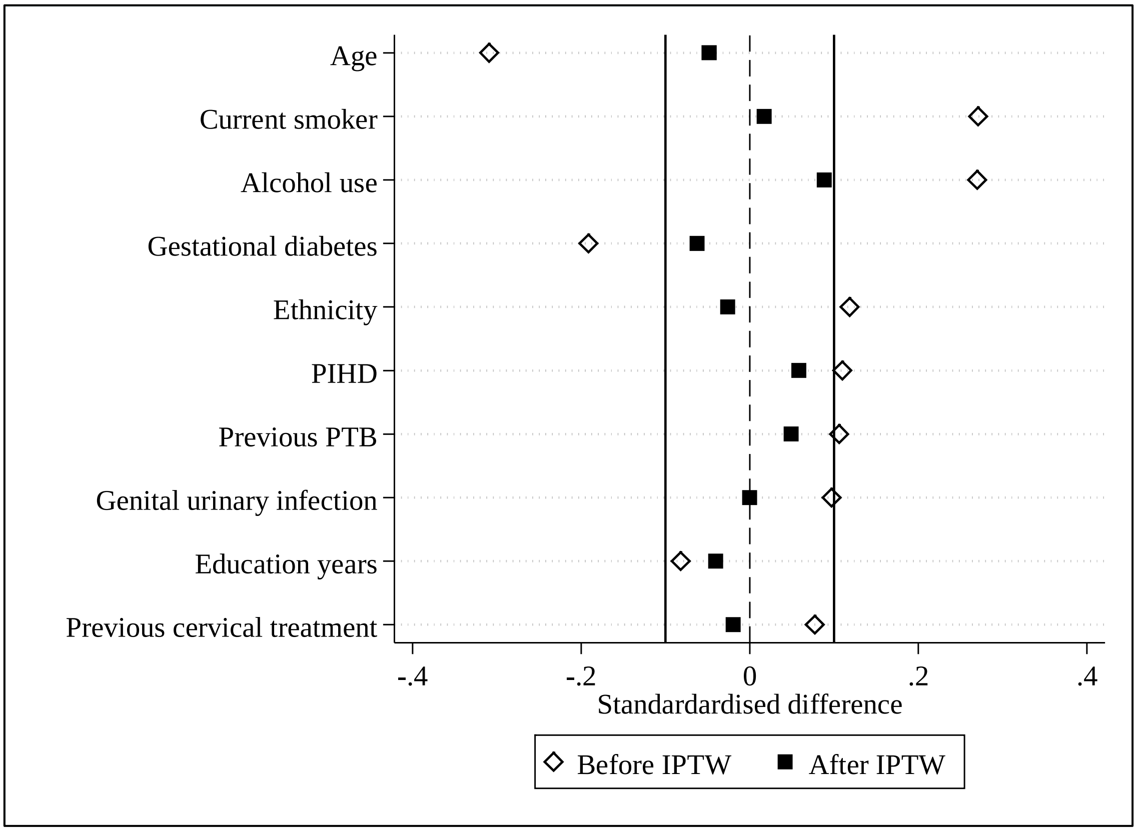

PTB: preterm birth; PIHD: pregnancy induced hypertensive disorders; IPTW: inverse probability treatment weighting

© 2021 Niyibizi J et al. *JAMA Network Open.*

eFigure 3. Kaplan-Meier Curves Presenting the Cumulative Incidence of Preterm Birth According to HPV16/18 Persistence During Pregnancy

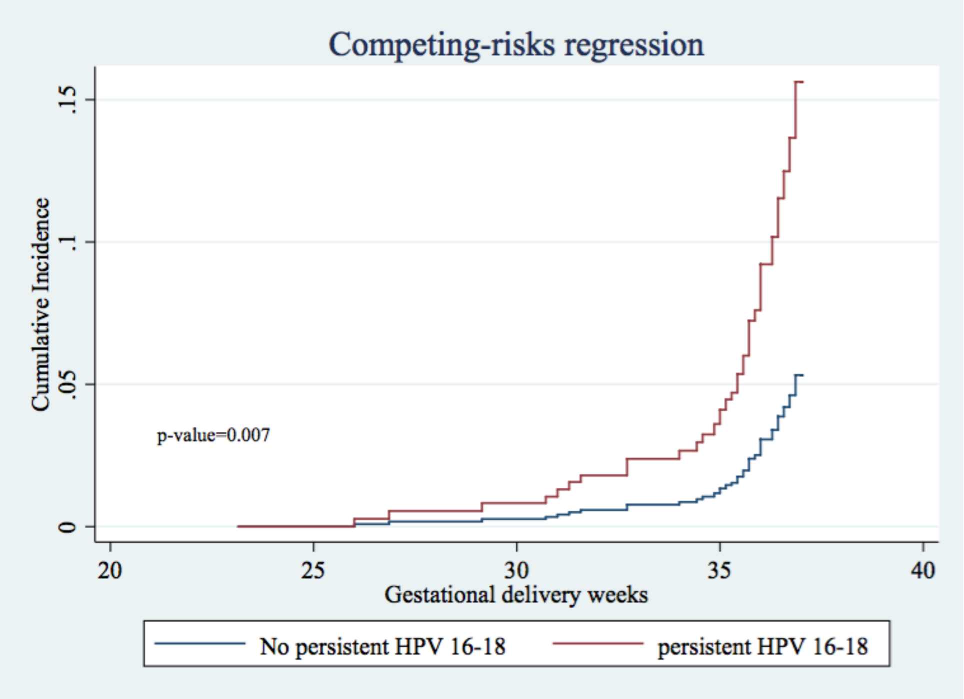

© 2021 Niyibizi J et al. *JAMA Network Open.*
