# Supplementary material for: Association Between Human Papillomavirus Infection Among Pregnant Women and Preterm Birth
Source: JAMA Netw Open. 2021 Sep 15;4(9):e2125308. doi: 10.1001/jamanetworkopen.2021.25308 (PMC8444026; doi:10.1001/jamanetworkopen.2021.25308)
Supplement: Supplement 2. — Nonauthor Collaborators. The HERITAGE Study Group Members [file jamanetwopen-e2125308-s002.pdf]

Supplemental Online Content: Nonauthor Collaborators

*Indicates required information. Only first name, last name, and suffix will appear in PubMed.

*Group Name(s): HERITAGE study group							
***First Name and Middle Initial(s)**	***Last Name**	***Suffix (eg, Jr, III)**	Academic Degrees	Institution	Location (city, state/province, country)	Role or Contribution, eg, chair, principal investigator	Group (if more than 1 Group listed in the byline) and/or Subgroup (eg, Steering Committee)
Helen	Trottier		MSc, PhD	Université de Montréal and Sainte-Justine University Hospital Center	Montreal, Qc, Canada	Principal Investigator	
Marie-Hélène	Mayrand		MD, PhD	Université de Montréal, Centre de Recherche du Centre Hospitalier de l'Université de Montréal	Montreal, Qc, Canada	Co-investigator	
François	Coutlée		MD	Université de Montréal, Centre de Recherche du Centre Hospitalier de l'Université de Montréal	Montreal, Qc, Canada	Co-investigator	
Patricia	Monnier		MD, PhD	Research Institute of the McGill University Health Center	Montreal, Qc, Canada	Co-investigator	
Louise	Laporte		Bacc.	Sainte-Justine University Hospital Research Center	Montreal, Qc, Canada	Study manager	
Joseph	Niyibizi		MD, PhD	Université de Montréal and Sainte-Justine University Hospital Center	Montreal, Qc, Canada	Research Assistant	
Monica	Zahreddine		MD, MSc	Sainte-Justine University Hospital Center	Montreal, Qc, Canada	Research Assistant	
Ana Maria	Carceller		MD	Sainte-Justine University Hospital Center	Montreal, Qc, Canada	Co-investigator	
Paul	Brassard		MD, MSc	Research Institute of the McGill University Health Center	Montreal, Qc, Canada	Co-investigator	
Jacques	Lacroix		MD	Université de Montréal and Sainte-Justine University Hospital Center	Montreal, Qc, Canada	Co-investigator	
Diane	Francoeur		MD	Université de Montréal and Sainte-Justine University Hospital Center	Montreal, Qc, Canada	Co-investigator	
Marie-Josée	Bédard		MD	Université de Montréal, Centre Hospitalier de l'Université de Montréal	Montreal, Qc, Canada	Site investigator	
Isabelle	Girard		MD	St-Mary's Hospital Center	Montreal, Qc, Canada	Site investigator	

Supplemental Online Content: Nonauthor Collaborators

*Indicates required information. Only first name, last name, and suffix will appear in PubMed.

***First Name and Middle Initial(s)**	***Last Name**	***Suffix (eg, Jr, III)**	Academic Degrees	Institution	Location (city, state/province, country)	Role or Contribution, eg, chair, principal investigator	Group (if more than 1 Group listed in the byline) and/or Subgroup (eg, Steering Committee)
François	Audibert		MD	Université de Montréal and Sainte-Justine University Hospital Center	Montreal, Qc, Canada	Site investigator	
William	Fraser		MD, MSc	Université de Sherbrooke, Centre de recherche du Centre Hospitalier Universitaire de Sherbrooke	Sherbrooke, Qc, Canada	Co-investigator	
